# Correlates of Sexual Risky Behaviours, HIV Testing, and HIV Testing Intention among Sexually Active Youths in Northern Tanzania

**DOI:** 10.24248/eahrj.v5i2.666

**Published:** 2021-11-15

**Authors:** Bernard Njau, Grace Mhando, Damian Jeremiah, Declare Mushi

**Affiliations:** a Amana Regional Referral Hospital, Dar-es-Salaam, Tanzania; b Kilimanjaro Christian University College, Kilimanjaro, Tanzania; c Kilimanjaro Christian Medical Centre, Kilimanjaro, Tanzania

## Abstract

**Background:**

HIV testing services are important entry-point into the HIV cascade to care and treatment in order to slow down the spread of HIV infection. Over half of all new HIV infections in Sub-Saharan Africa occur among young people under the age of 25, particularly women. The study aimed to determine factors influencing young people's decision to undergo HIV testing services in Northern Tanzania.

**Methods:**

A total of 536 sexually active participants aged 15 to 24 years old completed a semi-structured questionnaire based on the Health Belief Model (HBM) and the Theory of Planned Behaviour (TPB).

**Results:**

Males compared to femaleparticipants were significantly younger at first age of sexual intercourse (15.4 vs. 16.7 years; *p = .001*). Out of 536 participants, 418(78%) reported inconsistent condom use, and 203/303(67%) were those practicing casual sex. Only, 189/536(35.3%) of the participants reported to have had an HIV-test. Age, socioeconomic status, perceived HIV severity, attitudes and social approval regarding testing and beliefs about testing procedures and perceived barriers to testing were significant predictors of HIV testing (R^2^ =.22). Age, unsafe casual sex, perceived severity, HIV-testing attitudes, self-efficacy, social approval, cues for actions and perceived quality of testing procedures were significant and positively related to HIV-testing intentions, while perceived barriers to testing were negatively related (R^2^ = .36).

**Conclusion:**

The integrated constructs of HBM and TPB provides a framework for identifying correlates of HIV testing behaviours and HIV testing intentions among sexually active youths. Future behaviour change interventions should focus on reduction of sexually risky behaviours, increasing perceived HIV severity, enhance positive attitudes and social approvals on testing, reduce misconceptions about testing procedures, alleviation of perceived barriers to testing and improve testing self-efficacy among sexually active youths in this setting.

## BACKGROUND

More than 25% of the world population is made up of adolescents (aged 10 to 19 years) and youth (aged 15 to 24 years).^1^Globally, 50% of HIV transmissions occur among young people aged 15 to 24 and between 5,000 and 6,000 youths are infected with HIV every day. Further, over 50% of all new HIV infections in Sub-Saharan Africa (SSA) occurs in this vulnerable group, with young women disproportionately affected.^2^

In the United Republic of Tanzania, HIV incidence and prevalence has declined and stabilised. Currently, the HIV incidence is estimated at 0.24% in adults aged 15 years and older (approximately 72,000 new cases of HIV infection). HIV prevalence has declined from 7.0% in 2003/04 to 4.9 % in 2017, and the incidence of HIV infection in the age group of 15 to 24 years is estimated at 0.07%, whereas the overall HIV prevalence is 1.4%.^3^ However, there exist age and gender differentials in HIV prevalence, which is3 times higher among young women aged between 20 and 24 years compared to young men in the same age group (3.4 % vs. 0.9 %).

HIV Testing Services (HTS) are important means of slowing down the spread of HIV infection. These are provided free at both health facilities and community settings. HIV Care and Treatment Centres (CTCs) are established in both public and private health facilities across the country.^4^

In spite of the scale-up of the HTS and the availability of free Antiretroviral Treatment (ART) countrywide, only 49.0% of youths aged 15 to 24 years self-reported having ever took HIV testing services and received their test results, according to a recent HIV Impact Survey conducted in 2016-2017, with young women (60.0 % vs. 37.9%) more likely to test for HIV compared to their male counterparts. Also, among HIV-positive young adults, 60.9% were unaware of-their HIV status, with higher proportions among young men (64.9% vs. 59.4%) compared to their female counterparts.^3^

Comparatively to studies among adult populations in SSA, studies based on behavioural change models among adolescents and youth are rather sparse. Existing literature includes studies among youths^5^ at high-risk populations^6^ in Kenya, young school teachers in Tanzania,^7]^ and young women attending antenatal care in Ethiopia.^8^ Significant predictors of HIV testing intentions among 13 to 24 year olds includes; HIV knowledge, substance use, depression and social support.^5^

Several studies on factors associated with HIV testing and intentions among adolescents and youth conducted in Tanzania focused mainly on Knowledge, Attitudes and Practices (KAP), and not behavioural change models such as the Theory of Planned Behaviour(TPB),^9^ and the Health Belief Model(HBM).^10^ This study used a theory-based analysis of HTS decision-making among young people in Moshi, northern Tanzania. The conceptual framework for this study was the integrated constructs of the TPB^9^ and the HBM-the Integrated Behaviour Model (IBM).^10^ Using the IBM will provide a useful framework to investigate the correlates of HIV testing behaviours and HIV testing intentions among sexually active youths. Also, the study findings will add knowledge to the literature and inform HIV testing interventionist, Adolescent and Youth Sexual and Reproductive Health (AYSRH) policy makers on specific behaviours that may promote HIV testing and HIV testing intentions among out-of-school youths aged 15 to 24 years in northern Tanzania.

### Conceptual Framework

**FIGURE 1:**
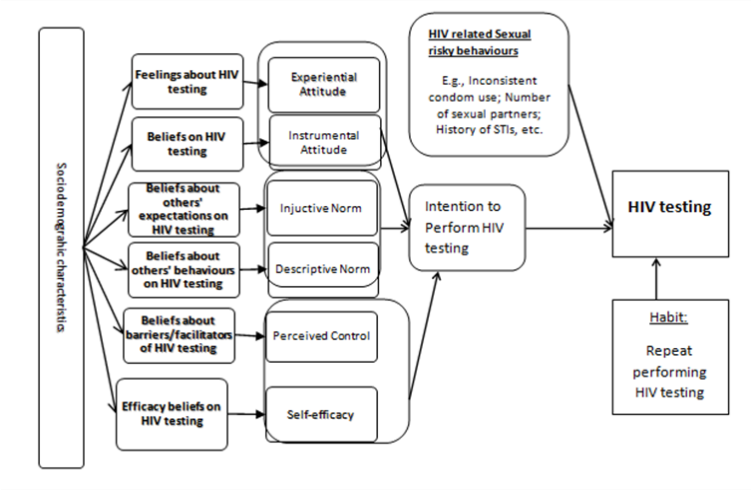
Summary of the Integrated Behaviour Model conceptual framework for sexual risky behaviours, HIV testing, and HIV testing intentions

Adapted from Glanz et al., 2008

Both HBM and TPB models have proven to be appropriate for understanding and predicting people's acceptance and uptake of health-related interventions, such as HTS.^9–12^ The IBM, which integrates constructs from HBM and TPB models posit that, people will take health-related actions if (i) they perceive themselves to be susceptible of contracting a specific illness (e.g., HIV), and if they perceive this specific illness as severe, (ii) they have a positive attitude towards the health behaviour (e.g., HTS), that is, if the perceived benefits of performing HIV testing outweighs the barriers or negative consequences of testing, (iii) they believe that they can successfully undertake HIV testing, (iv) they anticipate that their HIV testing behaviour will be approved by people in their social environment(e.g., parents, relatives, peers, religious leaders, etc.), and (v) they are ‘triggered’ to act.^13^ figure 1 below summarises the Integrated Behaviour Model conceptual framework for sexual risky behaviours, HIV testing, and HIV testing intentions.

## MATERIALS AND METHODS

### Inclusion/Exclusion Criteria

The inclusion criteria were age (15 years or older), outof-school, those who are willing to provide consent to participate, or from parents or guardian for those below 18 years, and able to coherently communicate in Kiswahilithe local language commonly used in the study settings.

### Study Area and Data Collection

The study was conducted in Moshi Urban, in Kilimanjaro Region, Northern Tanzania. Moshi Municipal Council is among the 7 districts in Kilimanjaro region of Tanzania. Other 6 districts are Rombo, Mwanga, Same, Moshi rural, Hai and Siha District Councils. Moshi Municipal Council has an estimated population of 184,292 mixed Muslim/or Christians, mainly from Chagaa and Pare tribes, residing in 21 ward administrative units, with an average household size of 4.0.^14^ Most of them engage in tourism, trading, and agro-economical activities. Moshi is also home to Mt. Kilimanjaro- the highest snow-capped mountain in Africa, a tourist circuitwith tourist hotels and bars and major national parks. At the time of data collection, the HIV prevalence among 15 years old and above in Kilimanjaro region was 2.2% (male=1.1%; female = 3.1%).^3^Also, there were 18 facilities providing HTS. In addition, 21 HIV Care and Treatment Centres(CTC) provide access to Antiretroviral Therapy (ART), in the study setting.^15^

A single population proportion sample size formula was used to calculate the sample size. Based on a HIV impact survey of 2016/2017^3^ which reported that 33.7% of young people aged 15 to 24 years old tested for HIV was used to calculate the sample size. The desired level of Confidence Interval (CI) at 95%, a margin error of 5% and non-response rate of 10 % were included in the formula as follows:

$$\text{N}={\text{Z}}^{2}\text{x}\;\text{P}\;\text{x}\;(100-\text{P})/{\text{E}}^{2}$$

where by N= Estimated Sample Size, Z = Standard Normal Deviation of 1.96^2^ corresponding to 95% CI, P = Proportion of outcome under study, and E = Marginal Error at 5%.^16^

The final sample size was derived at using the formula: Final sample size = Effective sample size/(1-non-responserate anticipated).

The minimum sample size was calculated as 343, and the anticipated non-response rate or dropout was 10% derived. The final minimal sample size = 343/(1–0.1) = 381 youth.

### Measurements

The questionnaire included the following variables. Unless indicated otherwise, we used 5-point Likert-scaled items to index the psychosocial variables.

#### Demographic Variables:

Demographic variables included; sex, age, relationship status, education level, religion, and media use. Participants were asked to report the presence of electronic devices in the household (e.g., radio, television, refrigerator, etc.). This variable served as a proxy measure for socioeconomic status.

#### Sexual Risk Behaviours:

Participants were asked about unprotected sex with their current or most recent steady partner, and/or casual partners, and lifetime condom use. Responses were dichotomised and coded as ‘no-condom’ or ‘condom’ depending on whether participants had engaged in unprotected sexual practices.

#### HIV Tests:

Participants were asked whether they had ever received an HIV antibody test. The expected response was 1= Yes; 2= No.

#### HIV-testing intentions:

Participant's intention to obtain an HIV-test in the next 6 months was indexed by 3 items -(e.g., “I intend to go for an HIV-test in the next 6 months”). The expected response ranged from 1= “most certainly not” to 5= “most certainly”. The Cronbach Alpha scale of reliability was = .90.

#### Perceived severity:

Perceived severity of HIV/AIDS was indexed by 3 items (e.g., AIDs is very severe illness), with answering categories varying from 1= “strongly disagree” to 5 = “strongly agree”. Because of extremely skewed distribution, items were combined to a binary index.

#### Perceived susceptibility:

Perceived susceptibility regarding HIV-infection was indexed by 3 items (e.g., “It is very likely that I will become infected with HIV/AIDS”), with expected response ranging from 1= “strongly disagree” to “5= “strongly agree”. This scale was reliable at α =.79.

#### Cues to action:

5 items addressing situations that may motivate people to go for a test; having had unsafe sex, marriage distrust in a partner, health status, and concurrent partners indexed Cues to action. Expected responses ranged from 1= “highly unlikely” to 5= “ highly likely”. The alpha scale of reliability was =.75.

#### Attitudes, beliefs, and barriers:

Attitudes towards HIV-testing was assessed by means of a general index (e.g., “Having an HIV-test is good/bad, and wise/unwise”; r= .06). A belief-based index consisting of 4 items addressing barriers to testing (e.g., expenses, fear of positive test results, losing hope and fear of stigma (α=.67), and 2 items addressing the reliability and confidentiality of test results(r=0.47). Answering categories for all belief-items ranged from 1= “highly unlikely” to 5= “highly likely”.

#### Social Approval:

Perceived social approval was indexed by means of 6 items addressing the social approval of significant others (e.g., parents, friends, relatives, partner, etc.) and institutions (e.g., church, school, health care, etc.). Expected responses range from 1= “strongly disagree” to 5= “strongly agree”. Participants indicating the absence of a social agent (e.g., having no sexual partner) received a score of 0 for the particular social agent. This scale was reliable at α = .87.

#### Self-efficacy:

Perceived self-efficacy regarding HIV-testing was assessed by 4 items (e.g., I am able to find out where I can go for an HIV-test), with answering categories ranging from 1= “most certainly not” to 5= most certainly.” The alpha scale of reliability was =.81. The Cronbach's alpha coefficient had a reliability index ranging from the lowest = .67 to the highest = .90. All of the reliability alphas were above the cut-off point of =. 60, a criterion for internal consistency of new scales.^17^

### Procedure

The interview guide was developed in English, back-and-forth translated into Kiswahili- a local language commonly used in Tanzania and subjected to a small-scale pilot (N= 10). After the potential participants and their parents (for those below 18 years) were informed of the study objectives. Using a local government register, a list of all out-of-school 15-24-years old was made and stratified by age groups and gender. Proportional sampling was used to select equal numbers of eligible males and females for participation in the study. 4 (2 males; 2 females) trained research assistants, with previous experience in conducting studies addressing sensitive topics (i.e., sexuality and HIV/AIDS), and members of a local youth organisation concerned with HIV/AIDS, of the same age group (15 to 24 years old) with the studyparticipants administered the interview guide to eligible participants after consenting. To ensure confidentiality, filled questionnaires were put in large brown envelopes after completion; no names of participants were registered, instead coded numbers were used to ensure anonymity. Data was collected between September and November 2016.

### Data Analysis

All statistical analysis was conducted using Scientific Package for Social Science(SPSS for Windows; SPSS, Chicago, IL, USA version 20). Reliability test was employed to address the internal reliability of scaled variables. We employed hierarchical logistic regression analysis to identify the psychological and socio-demographic correlates of HIV-testing behaviour. In the first block, we controlled for socio-demographic variables. In the second block, we entered the indexes for unprotected sexual activity. In the third block, we added cognitive variables to the regression equation. For the regression of HIV-testing intentions, we followed a similar procedure, albeit employing linear regression.

### Ethical Consideration

The study received ethical approval from Kilimanjaro Christian Medical College Research Ethics Committee(Ref No: 737/2016), and from local administrative officers for study implementation. The data collection procedure was explained to all participants on their voluntary participation and the right to withdraw from the study without any consequences. Data was collected in a private room within the local youth organisation offices to ensure confidentiality, and acquire independent and accurate responses. No names of participants were used on the questionnaire but coded numbers to ensure anonymity.

## RESULTS

### Characteristic of Respondents

A total of 1,183 participants aged 15 to 24 years old were recruited and 536 (45.3%) reported to have had sexual experiences. These sexually experienced young people are the sample of analysis and reported in this study. Of the 536 sexually experienced participants, 342(63.8 %) were aged 15 to 19 years old. The mean age was 19.4(SD=3.14), with women being significantly younger than men(18.9; SD= 2.98; 19.9; SD = 3.35; ***p = .000***). More than half, 295 (55%) of participants were single compared with 45% who were married. More than half, 279 (52.1 %) of the participants were able to read, and 303 (56.6 %) reported having a Catholic denomination. The respondents' characteristics are summarised in Table 1.

**TABLE 1:** Sociodemographic of Sexually Experienced Respondents (n=536)

Variables	Total N= 536(%)	Males n= 309(57.6)	Female n= 227(42.4)
**Age group (years)**			
15–19	342(63.8)	210(61.4)	132(38.6)
20-24	194(36.2)	99(51.1)	95(48.9)
Mean age (SD)	19.4(3.14)	19.4(3.35)	18.9(2.98)
**Marital status**			
Single	295(55)	177(60)	118(40)
Married	241(45)	109(45.2)	132 (54.8)
**Ability to read**			
Able to read	279(52.1)	148(53.1)	131(46.9)
Read with difficulty/not at all	257(47.9)	141(54.8)	116(45.2)
**Occupation**			
Employed	229(42.7)	146(63.8)	83(36.2)
Unemployed	307(57.3)	117(38.2)	190(61.8)
**Religion**			
Muslim	65(12.2)	44(67.7)	21(32.3)
Protestant	168(31.2)	97(57.7)	71(42.3)
Catholic	303(56.6)	180(59.4)	123(40.6)
Row percentages			

### Sexual Activity, Condom use, and HIV-testing

On average, participants were nearly 16 years old when they had their first sexual intercourse. Males were significantly younger when they had their first sexual experience than their female counterparts (15.4 vs. 16.7 years, respectively; F(1,447)= 21.6, *p=.001*). Male participants reported having had their first sexual intercourse with-younger partners (mean age of female partner =14.8), whereby female participants indicated to have had their sexual debut with older sexual partners (mean age of male partner = 20.3). Of 227 female participants, 177(77.8%) compared to 200/309(64.7%) male participants reported being in a steady relationship. Out of 309 male participants, 179 (58%) and 124/227(55%) of the female participants indicated to have had casual sex in the past 3 months (i.e., sexually active) prior to the study. Out of 536 participants, 418(78%) reported inconsistent condom use, and 203/303 (67%) who had casual sex reported inconsistent condom use. More than a third, 189/536 (35.3%), of the participants, reported having had an HIV-test (men = 35.8 % vs. women= 34.6 %). There were no significant gender differentials in condom use and HIVtesting.

### Correlates of HIV-Testing

Multivariate hierarchical logistic regression analysis revealed that age and socioeconomic status (Model 1), accounted for a small proportion of the variance in testing behaviour (R^2^ =^.^ 04). Adding indicators for unsafe sexual activity (Model 2) almost doubled the predictive quality of the model. The results indicate that younger participants with a relatively low economic status, and reporting unsafe sex with casual sex partners are more likely to report having ever been tested for HIV. Inclusion of psychosocial variables (Model 3) increased the proportion of the variance in HIV testing behaviour (R^2^ = .22). These findings indicate that predictors for engaging in HIV-testing include: younger age, low socio-economical status, higher scores on perceived HIV severity, attitudes and social approval regarding testing and beliefs about testing procedures, and low scores on perceived barriers to testing. (Table 2)

**TABLE 2:** Correlates of HIV Testing: Hierarchical Multivariate Logistic Test

	Model 1		Model 2		Model 3	
**Variable**	**OR**	**95%CI**	**OR**	**95%CI**	**OR**	**95%CI**
Age	1.09	1.02–1.16	1.09	1.02–1.17	1.11	1.03–1.12
Gender	.91	.61–1.36				
Religion						
Catholic	1.00					
Muslim	.72	.42–1.23				
SES						
High	1.00		1.00		1.00	
Mild	.64	.34–1.21	.68	33–1.38	.44	.20–.92
Low	.61	.38–.96	.56	.33–0.93	.51	.30–.87
Unprotected sex						
With steady partner			0.68	1.14–3.05		
With casual partner			1.95	1.20–3.16	1.04	.65–1.64
Susceptibility					.99	.91–1.07
Severity					1.96	1.10–3.51
Attitude					1.22	.99–1.50
HIV test quality					1.23	1.05–1.44
Perceived barriers					.91	.85–.98
Social Approval					1.05	1.00–1.10
Self-efficacy					.99	.91–1.10
Cues to Action					1.01	.94–1.08
Nagelkerke R2	.04		.07		.22	
N	473		375		428	

### Correlates of HIV-Testing Intentions

In the linear regression analyses of HIV-testing intention on socio-demographic variables, only age was found to significantly account for the variance of HIV testing intention (Model 1). The findings indicated that older participants were more likely to report favourable intentions towards HIV-testing. Inclusion of the previous history of HIV testing and the indicators for unsafe sexual activity to the regression model 2 increased the variance for HIV-testing intentions to 5.2%, indicating that previous history of HIV testing, and unsafe casual sexual as significant predictors. Addition of psychosocial variables to the regression model 3, revealed that attitude towards HIV-testing, testing self-efficacy, social approval, cues to actions, and perceived quality of testing procedures were significant and positively associated with HIV testing intentions. Furthermore, perceived barriers to HIV-testing were negatively associated with testing intentions. Both previous history of HIV-testing and unsafe casual sex which were significant in model 2 were deleted from model 3. The remaining variables (age and psychosocial variables) accounted for 36.3% of the variance in testing intentions. (Table 3)

**TABLE 3:** Correlates of HIV Testing Intentions: Hierarchical Multivariate Linear Test

	Model 1		Model 2		Model 3	
Variable	β	P	β	P	β	P
Age	.10	.023	.11	.012	.11	.004
Gender	-.07	.107				
Religion						
Catholic						
Muslim	.02	.602				
Protestant	.04	.358				
SES						
High						
Mild	−.05	.301				
Low	.05	.296				
Past HIV testing			.18	.000	.04	.338
Unprotected sex						
With steady partner			.03	.665		
With casual partner			−.13	.003	−.04	.237
Susceptibility					.11	.005
Severity					−.02	.546
Attitude					.28	.000
HIV test quality					.11	.007
Perceived barriers					−.13	.000
Social Approval					.12	.006
Self-efficacy					.13	.005
Cues to Action					.13	.003
R2	1.4%		5.4%		36.3%	

## DISCUSSION

The aim of this study was to determine the correlates of sexual risky behaviours, HIV-testing and HIV testing intentions among sexually active youths aged 15 to 24-years old in Moshi, Kilimanjaro region of northern Tanzania. The findings demonstrate that majority of sexually active youth urbanites continue to practice unsafe sex. Many reported to practice unsafe sex in steady relationships, and more-worrying-when having casual sex. These findings are consistent to those reported by studies in other Sub-Saharan African countries.^5,18,19,21^ Also, the study findings indicate that about one-third of sexually active young people have been engaged in HIV testing. This observation concurs with existing evidence that there is an increase of youth participation in HTS, however, the turn up is still low in comparison to the good availability of HTS in the study setting. The observation that both sexual reduction and participation in HTS is still rather uncommon among a large proportion of sexually active young people underlines the strong need for ongoing efforts to motivate youth to engage in HIV-prevention intervention.^5,18,21,22^

This study suggests that activities aimed at motivating young people to participate in HTS should include; risk reduction communication, attitude change, norm setting and facilitation. This concurs with findings from other settings suggesting that behaviour change interventions to promote HIV-testing should go beyond increasing HIV knowledge and health-related risks, and should address test confidentiality and the fear for positive test results and social exclusion.^5,7,18,21,22^

In our study, we have used an integrative conceptual framework to determine the socio-demographic, behavioural and social-cognitive predictors of HIV-testing behaviours and intentions. The findings show that this conceptual framework was not that successful in predicting HIV-testing behaviour; the final model accounted for only 16% of the variance in testing behaviour. Socioeconomic status and perceived severity were identified as the most important predictors, but these variables were conceptually not the strongest. HIV-testing behaviour was associated with perceived susceptibility, attitudes and beliefs regarding testing, although odds ratios were rather low. To enhance a deeper understanding of testing uptake, it would be worthwhile when HTS include the identification of predictors that motivate youths to engage in testing by using a standardised interview protocol.^5–8,18,28^

Superior experimental study designs such as clinical trials, and/or Discrete Choice Experiments (DCEs) with a multi-site set-up, would tremendously improve our understanding in HIV-testing behaviours and intentions among young people.

According to Ajzen^9^, the HBM and TPB models are developed to predict future behaviour rather than to understand past behaviours and this is in line with respect to the prediction of uptake of future HIV testing in our study. In this study, the predictive quality of the model was far better with 36% of variance accounting for attitudes, beliefs about testing, social approval, self-efficacy, anticipated barriers, cues to action and age as major predictors.

This study reported conflicting results regarding sexual risk behaviours, HIV-testing and HIV-testing intentions. Participants who reported practicing unsafe casual sex were more likely having been tested, but unsafe casualsex was negatively related to testing intentions. Additionally, whereby perceived HIV severity was positively related to previous testing behaviours, perceived HIV risk was positively related to HIV-testing intentions. Also, additional analyses revealed that perceived susceptibility was unrelated to sexual risk-taking behaviours. The most plausible explanation to this observation regarding the association between risk-taking behaviours, risk perceptions, and screening could be due to reflecting complex psychological processes including risk denial and emotion-based coping.^18,22^ This warrants for future mixed-methods longitudinal studies to unravel this process.

Regarding HIV-testing intentions, a critical concern is the inability to translate intentions into actual behaviour. Some studies have suggested that for various reasons, individuals may not execute their plan to take HIV test.^7,8,18^ Sheeran ^29^, however, concluded in his meta-analysis of meta-analyses that intentions on average do predict 28% of the variance in future behaviour and that individuals with attitudinal controlled intentions-similar to this study generally have stronger intention-behaviour correlations than individuals with normatively controlled intentions.^29^ Nevertheless, a longitudinal study is warranted to further identify intention-behaviour gaps regarding HIV-testing among young people. The aim will be to identify the individual and community level predictors which are highly contextual to a complex social setting that need to be targeted to reduce these gaps and to identify intervention techniques to strengthen intention-behaviours for HIV-testing.^22^

Despite these study findings based on a relatively large sample of young people, it is imperative to consider some limitations. As a cross-sectional study design, it is not possible to draw conclusions about causality of any of the identified associations. This study is based upon self-reporting of previous sexual risk behaviours, HIV-testing behaviours, and HIV testing intentions. Reporting bias could be a limitation with over-reporting or under-reporting of the risk-taking behaviours and HIV testing. Longitudinal study designs would be the most ideal approach in studying the correlates of HTS, although it is more expensive and labour intensive. Generalisability of the result to other young people in Tanzania may be another limitation because it was conducted among outof-school population aged 15 to 24 years, in an urban setting of Moshi and may not be applicable to other settings, or populations. Another limitation could be the use of the HBM, a cognitive-based model, which is limited in assessing the emotional components of behaviour. Also, caution is warranted regarding the reliability and internal validity of the data because some item-level analyses were limited to moderate internal reliability of scales (barriers to testing; α =0.67). Finally, this study did not address the impact of structural and environmental factors that may facilitate or impede scaling up of participation in HTS.

While the decision to take an HIV test is usually an individual's choice, future research should determine the influence of other factors, such as, gender differentials and economic inequalities, HIV testing options (e.g., HIV self-testing), client preferences and selectivity of HTS, stigma and discrimination related with HTS.^5–7,18,21,22,25,30–33^

## CONCLUSION

The study findings indicate that sexually experienced out-of-school-youths in this study setting practice sexual risky behaviours, including unprotected sex, and multiple sexual partnerships with low uptake of HTS. HIV interventionist and HIV policymakers should focus on designing theory-based behaviour change interventions with focus on motivating and facilitating adolescents and youths regarding sexual risk reduction, increasing perceived HIV severity, enhancing positive attitudes and social approvals on testing, reducing misconceptions about testing procedures, alleviation of perceived barriers to testing and improving testing self-efficacy among sexually active youths.^34^
